# Novel and recurrent genetic variants of *VHL*, *SDHB*, and *RET* genes in Chinese pheochromocytoma and paraganglioma patients

**DOI:** 10.3389/fgene.2023.959989

**Published:** 2023-03-03

**Authors:** Chong Li, Jingyi Li, Chao Han, Ting Wang, Lixia Zhang, Zhifang Wang, Tingting Wang, Lijun Xu, Guangzhao Qi, Guijun Qin, Xialian Li, Lili Zheng

**Affiliations:** ^1^ Department of Endocrinology and Metabolism, The First Affiliated Hospital of Zhengzhou University, Zhengzhou, China; ^2^ Department of Plastic Surgery, Beijing Tiantan Hospital, Capital Medical University, Beijing, China; ^3^ Department of Pharmacy, The First Affiliated Hospital of Zhengzhou University, Zhengzhou, China

**Keywords:** pheochromocytoma, paraganglioma, VHL, SDHB, RET

## Abstract

**Background:** Pheochromocytoma and paraganglioma (PPGL) are rare neuroendocrine tumors arising from chromaffin cells in the adrenal medulla and extra-adrenal ganglia, respectively. The study was aimed to investigate the clinical and genetic characteristics of 22 individuals from six families.

**Methods:** The medical records of six PPGL probands who presented to our hospital between 2016 and 2021 were retrospectively studied. DNA isolated from the probands was analyzed using whole exome sequencing. The identified genetic variants were confirmed by Sanger sequencing and undergone bioinformatic analysis.

**Results:** Six different genetic variants in the six probands were identified, respectively, of which three were novel. A novel von Hippel-Lindau *(VHL)* variant, c.602T>C (p.L201P), in exon 3 was found. Two novel genetic variants in *SDHB* (succinate dehydrogenases subunit B), c.423 + 1 G>T and c.662A>G (p.D221G), were identified. Two recurrent genetic variants of *VHL*, c.C284G (p.P95R) and c.558_560AGAdel (p.186Edel), and one in *RET* (ret proto-oncogene), c.1901G>A (p.C634Y), were also found. The ClinVar accession number for the present variants are SCV002028348, and SCV002028352 to SCV002028361.

**Conclusion:** Genetic variants in *VHL*, *SDHB* and *RET* were identified in Chinese PPGL patients, which contributed to the knowledge of the genetic etiology and clinical outcome of these tumors.

## Introduction

Pheochromocytoma (PCC) and paraganglioma (PGL) (PPGL) are rare neuroendocrine tumors which arise from chromaffin cells in the adrenal medulla and extra-adrenal ganglia of the autonomic nervous system, respectively. PPGL could produce catecholamines such as epinephrine, norepinephrine, and dopamine leading to clinical presentation of hypertension, headache, diaphoresis, palpitations and so on (Lenders et al., 2020). PPGL are estimated to occur in approximately 0.4 to 9.5 per 1 million people per year. In addition, about 0.1% of patients with hypertension could be attributed to PPGL (Guilmette and Sadow, 2019; Aygun and Uludag, 2020a; Aygun and Uludag, 2020b). In addition to the neural crest-derived endocrine cellular tumor, other neoplasms including hemangioblastoma in the retina and central nervous system and renal cell carcinoma could be found in the same individual with other kinds of syndromes such as multiple endocrine neoplasia type 2 (MEN 2), Von Hippel-Lindau syndrome (VHL), neurofibromatosis type1 (NF1) and familial pheochromocytoma/paraganglioma syndromes (Antonio et al., 2020). The heterogeneity of clinical symptoms and tumor locations and pathogenesis in PPGL complicates the diagnosis and treatment of patients.

The majority of PPGL were sporadic, while about 40% patients had a germline mutation in the susceptibility genes including *VHL*, rearranged during transfection (*RET*), *NF1*, succinate dehydrogenases subunits (*SDHA*, *SDHB*, *SDHC*, *SDHD*, and *SDHAF2*), hypoxic inducible factor 2 alpha (*HIF2A*), prolyl-hydroxylase domain protein 1 (*PHD1*), *PHD2*, fumarate hydratase (*FH*), myc-associated factor (*MAX*) and so on. The Cancer Genome Atlas (TCGA) program had divided PPGL with these mutations into three groups (Fishbein et al., 2017). Although imaging tests such as magnetic resonance imaging (MRI), computed tomography (CT), positron emission tomography (PET) and specialized nuclear medicine imaging such as a metaiodobenzylguanidine (MIBG) scan could detect the adrenal incidentalomas (Sarkadi et al., 2021), genetic testing on the susceptibility genes could find a germline or somatic mutation, which might be a useful hint for the personalized treatment by targeted therapy.

The characterization by different genotype-phenotype relationships in PPGL was largely restricted to European populations (Cascón et al., 2009; Cascón et al., 2013; Casey et al., 2017; Rijken et al., 2018). A recently study compared Chinese and European PPGL patients and found higher frequencies of *HRAS* (HRas Proto-Oncogene, GTPase), *FGFR1* (fibroblast growth factor receptor 1) and *HIF2A* mutations in Chinese than Europeans but higher frequencies of *NF1*, *VHL*, *RET*, and *SDH* mutations in Europeans than Chinese (Jiang et al., 2020).

VHL and SDH complex share a hallmark feature of Pheochromocytoma, but there are some differences between them. PPGL caused by mutation of *VHL* tumor suppressor gene is called VHL disease/syndrome Type two, occurring with CNS hemangioblastoma, renal or pancreatic cysts, renal carcinoma and exodermic cystadenoma. SDH complex, also called hereditary PPGL, is caused by mutation in the *SDH* (*SDHD*, *SDHB*, and *SDHC*) genes. Most patients belonged to the sporadic presentation tumors. The symptoms and characterization differences between VHL and SDH complex are shown in Supplementary Table S1.

Therefore, more detailed clinical manifestation and molecular genetics analyses of PPGL among different world populations are needed to be performed. The aim of the present study was to characterize the clinical presentation and genetics of six Chinese PPGL patients and to evaluate the potential function of the identified genetic variants using online software.

## Methods

Six probands with pathological diagnosis of PPGL and their family members were recruited in this study. The present study protocol was conducted in accordance with the Declaration of Helsinki and approved by the Ethics Committee of The First Affiliated Hospital of Zhengzhou University, Zhengzhou, China. Written informed consents were obtained from all subjects. PPGL tumor samples from every proband were collected immediately after the surgery performed in the Department of Urology Surgery of The First Affiliated Hospital of Zhengzhou University. Peripheral blood from six probands and their family members were collected when they were in hospital. All samples were immediately frozen in liquid nitrogen and stored at −80°C.

Genomic DNA was extracted from the PPGL tumor samples, which was then used for the generation of exome libraries by the Illumina TruSeq DNA Sample Prep kit, following the manufacturer’s instruction. The targeted gene exons and adjacent splicing regions (about 50 bp) were captured by probe hybridization and enriched. The captured coding sequences with the Agilent SureSelect All Exon kit-v4 were sequenced on the Illumina HiSeq 2500 platform with an average sequencing depth of ×100 for the targeted regions. Reads that did not meet the quality control requirements were first removed from the original sequencing data, and then BWA (Burrows-Wheeler Alignment) software was used to compare with the hg19 version of the human genome reference sequence provided by UCSC (University of California, Santa Cruz). SNP (single nucleotide polymorphism) and InDel (insertion and deletion) variations were identified by GATK’s (Genome Analysis Toolkit) HaplotypeCaller. Data interpretation rules refer to Americal College of Medical Genetics and Genomics (ACMG) Classification Standards and Guidelines for Genetic Variation. Only known genetic variants clearly associated with inherited diseases were analyzed, and some genes whose functions and pathogenicity were not clearly defined were excluded from the analysis. Common benign polymorphic variants, synonymous variants, and intron variants that do not affect mRNA splicing would not be included in the data analysis unless the pathogenicity is reported in the literature or recorded in the database.

Genomic DNA was extracted from the peripheral blood samples, which was then used for the Sanger sequencing to validate the genetic variants identified by WES. Briefly, the DNA sequences containing the coding region of *VHL*, *SDHB*, and *RET* gene were amplified by polymerase chain reaction (PCR) sequenced by 3730XL sequencer.

The functional consequences of missense variants were predicted through a panel of online computational algorithms including SIFT (http://sift-dna.org) (Ng and Henikoff, 2003), PolyPhen2 (http://genetics.bwh.harvard.edu/pph2/) (Adzhubei et al., 2010), PROVEAN (http://provean.jcvi.org/index.php) (Choi and Chan, 2015), and MutationTaster (http://mutationtaster.org/) (Schwarz et al., 2010). In addition, splice acceptor and splice donor were assessed by Combined Annotation Dependent Depletion (CADD) (https://cadd.gs.washington.edu/) (Rentzsch et al., 2019) and MutationTaster.

We also investigated the coding variants of *VHL*, *SDHB*, and *RET* genes in CMDB in the present study. The Chinese Millionome Database (CMDB), a large-scale Chinese population sequencing project, investigated 141,431 whole genome sequences from Chinese women who took a non-invasive prenatal testing (Liu et al., 2018). The allele frequencies of diverse genes in Chinese provided an important reference database for research on the disease susceptibility genes and drug response pharmacogenes in China (Qi et al., 2020a; Qi et al., 2020b; Qi et al., 2021; Qi et al., 2022). We had deposited the identified variants to the ClinVar database with accession numbers of SCV002028348—SCV002028352 and SCV002028361.

## Results

### Proband 1

**FIGURE 1:**
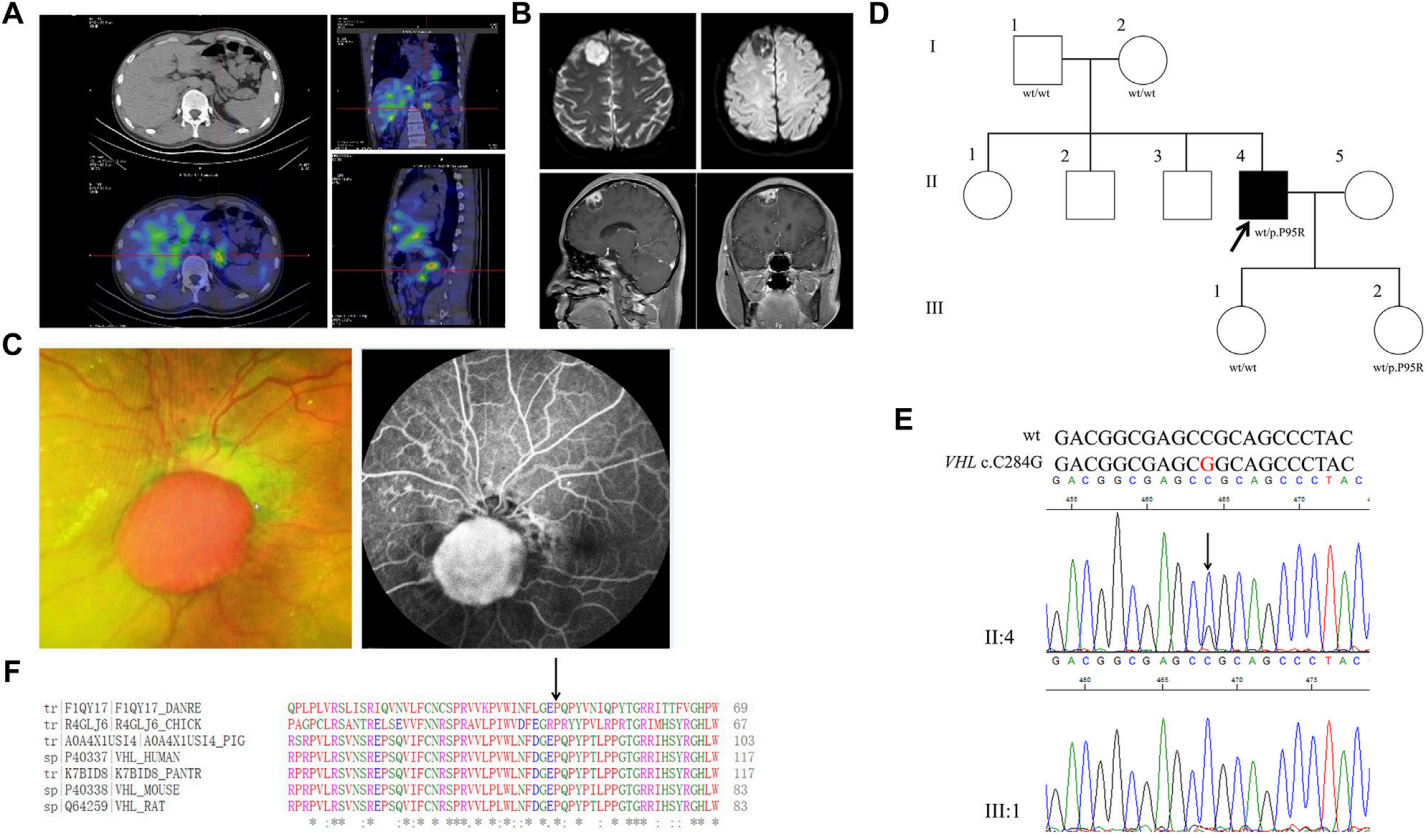
Clinical and genetic characteristics of Proband 1. **(A)** 131I-MIBG SPECT/CT imaging in a 30-year-old male patient with PPGL showing a focus of radioactivity distribution in the left adrenal gland. CT (upper) and SPECT/CT (bottom) transaxial sections are shown in the left column, while coronal section in the upper right and sagittal in the bottom right. **(B)** Dilated funduscopic examination of the patient’s left eye showing an orange nodular lesion (left) in the surface of optic disc and fluorescein angiography showing leakage and marked hyperfluorescence of the nodule, indicative of retinal hemangioblastoma (right). **(C)** DWI (upper) and T1WI (bottom) in the Proband 1with meningioma. DWI, Diffusion-weighted imaging; T1WI, T1-weighted image. **(D)** Pedigree of the Proband 1 family with PPGL. Squares and circles indicate males and females, respectively; Roman numerals indicate generations; and Arabic numerals indicate individual family members. Each member’s *VHL* genotype is shown below the squares and circles. WT, wild type. P, proline; R, arginine. **(E)** DNA sequencing data of an unaffected female (III:1) and the Proband 1 (II:4) with the heterozygous variant of *VHL* c.C284G. WT, wild type. **(F)** Multiple protein sequence alignment of seven species for VHL p.P95 (arrow). An asterisk (*) indicates positions that are 100% conserved in the alignment, and a colon (:) indicates conservation between groups of strongly similar properties, whilst a period (.) indicates conservation between groups of weakly similar properties. The residues in the alignment are colored to indicate their physicochemical properties. Red indicates small and hydrophobic amino acids, blue indicates acidic residues, magenta indicates basic and green denotes hydroxyl/sulfhydryl/amine residues.

The 30-year-old male was hospitalized for episodic palpitations, headache, and blurred vision for 19 years. Nineteen years ago, he was found to have a right adrenal occupying lesion (5*2.6*3.5 cm) by ultrasonography. After the right adrenal pheochromocytoma excision he had a normal blood pressure and a better vision. Fifteen years ago, a left adrenal occupying lesion was found and excised. Four years ago, retroperitoneal hypoechoic nodules next to the great vessels were found and multiple occupancies in the left adrenal and pheochromocytoma between the vena cava and portal vein were identified by abdominal enhanced CT (Figure 1A). After laparoscopic resection of ectopic pheochromocytoma, para-aortic paraganglioma was identified. Blood monoamine neurotransmitters were normal (Table 1). Left eye retinal hemangioblastoma was found by the fundus fluorescein angiography (Figure 1B). Right frontal lobe occupancy (2.2*2.0*0.7 cm) was found by brain MRI (Figure 1C). After resection of the right intracranial occupancy, meningioma was identified by immunohistochemistry.

**TABLE 1:** Concentrations of catecholamines in the six patients.

Laboratory test	Proband 1	Proband 2	Proband 3	Proband 4	Proband 5	Proband 6	References value
urinary epinephrine (μg/24 h)	16	12	12	246	110	223	0–20
urinary norepinephrine (μg/24 h)	54	35	30	293	465	427	0–50
plasma epinephrine (nmol/L)	0.2	<0.12	<0.12	NA	<0.12	51.46	0–0.34
plasma norepinephrine (nmol/L)	3.35	0.15	2.27	NA	3.76	108.05	0–5.17
plasma metanephrines (nmol/L)	<0.12	<0.12	<0.12	<0.12	<0.12	14.28	0–0.42
plasma normetanephrines (nmol/L)	0.33	<0.12	<0.12	<0.12	0.48	14.71	0–0.71

NA, not available.

Through WES of the genomic DNA of the PPGL sample of proband 1, a *VHL* variant, c. C284G, in exon 1 was found with the amino acid proline in codon 95 (CCG to CGG) substituted by arginine (p.P95R) (Figure 1D). We had not found this variant in the database of gnomAD (Genome Aggregation Consortium), CMDB or ClinVar, but it had been recorded in HGMD (Human Gene Mutation Database). The genomic DNA from meningioma tissue and blood were used for the Sanger sequencing, and the variant c.C284G was identified (Figure 1E). Parents of proband 1 and his two daughters were sequenced for the c.C284G variant by Sanger sequencing and only his younger daughter had this variant, which demonstrated that *VHL* c.C284G was a *de novo* mutation for proband 1. His parents, older sisters, two brothers and two daughters were all healthy by 5 March 2020. By PolyPhen-2, this variant was predicted to be probably damaging with a score of 1.00. In addition, this variant was predicted to be deleterious with a PROVRAN score of −3.89; MutationTaster and SIFT also demonstrated it to be disease causing. Aligning the VHL protein sequences with the Clustal Omega program demonstrated that the proline at codon 95 of the VHL protein was conserved among diverse species (Figure 1F). In total five missense and one stop codon variants of *VHL* were found in CMDB, which were all rare with a minor allele frequency less than 0.01 (Supplementary Table S2).

### Proband 2

**FIGURE 2:**
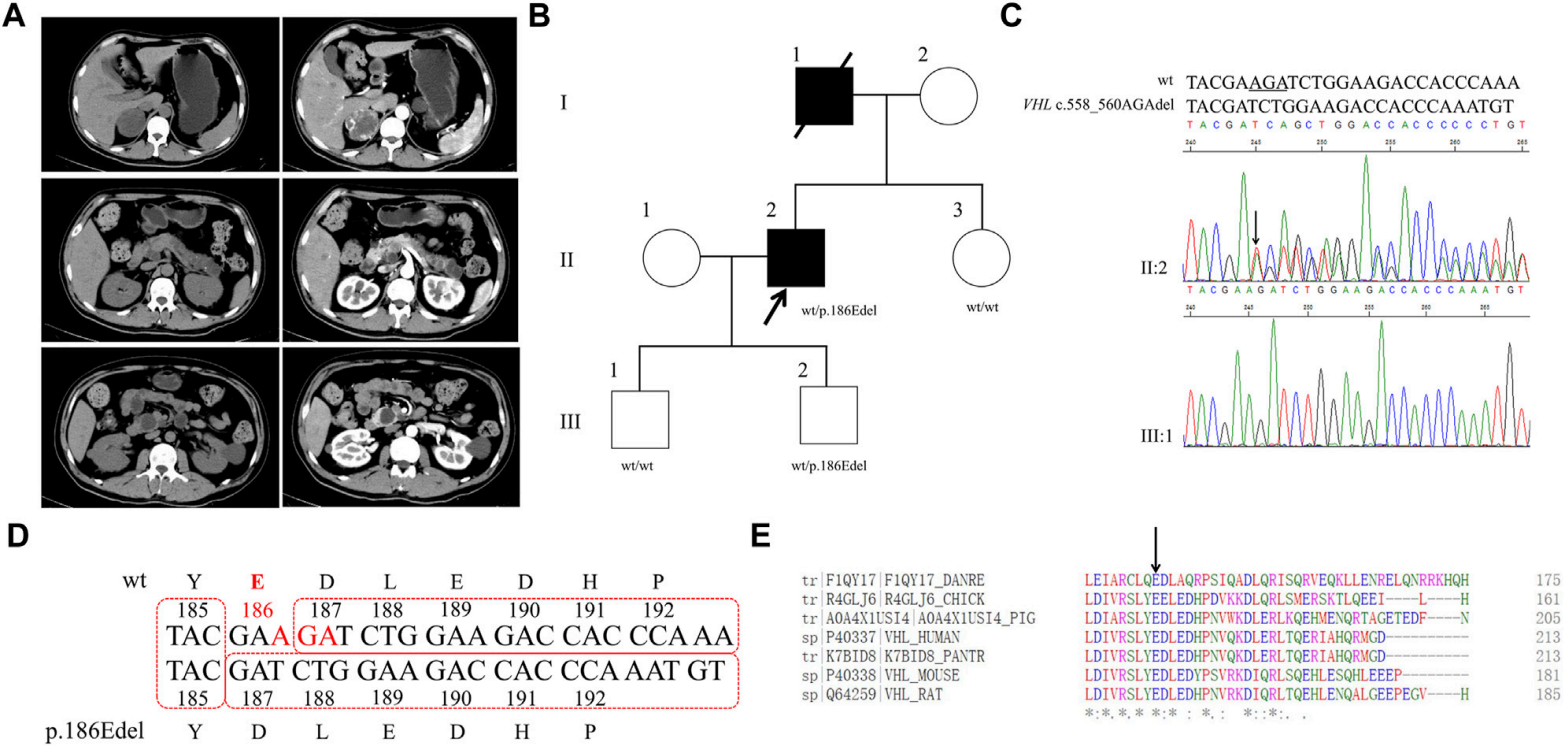
Clinical and genetic characteristics of Proband 2. **(A)** CT (left) and contrast-enhanced CT scan (right) imaging in a 33-year-old male patient with PPGL showing right adrenal occupancy (upper), pancreatic polycysts (middle), and kidney carcinoma (bottom). **(B)** Pedigree of the Proband 2 family with PPGL. Squares and circles indicate males and females, respectively; Roman numerals indicate generations; and Arabicnumerals indicate individual family members. Each member’s *VHL* genotype are shown below the squares and circles. Wt, wild type. E, glutamic acid. Del, deletion. **(C)** DNA sequencing data of an unaffected male (III:1) and the Proband 2 (II:2) with the heterozygous variant of *VHL* c.558_560AGAdel. Wt, wild type. Del, deletion. **(D)** The deletion of glutamic acid in codon 186 of *VHL* resulting from *VHL* c.558_560AGAdel. Wt, wild type. Y, tyrosine; E, glutamic acid; D, aspartic acid; L, lysine; H, histidine; P, proline; del, deletion. **(E)** Multiple protein sequence alignment of seven species for VHL p.186E (arrow). An asterisk (*) indicates positions that are 100% conserved in the alignment, and a colon (:) indicates conservation between groups of strongly similar properties, whilst a period (.) indicates conservation between groups of weakly similar properties. The residues in the alignment are coloured to indicate their physicochemical properties. Red indicates small and hydrophobic amino acids, blue indicates acidic residues, magenta indicates basic and green denotes hydroxyl/sulfhydryl/amine residues.

A 33-year-old male was hospitalized for hyperglycemia for half a year, hypertension for 4 months, and hematuresis for 20 days. Through abdominal CT, pancreatic polycysts, right adrenal occupancy, and kidney carcinoma were found (Figure 2A). Right renal clear cell carcinoma, left renal clear cell carcinoma, and right adrenal pheochromocytoma were diagnosed. After the transurethral resection of bladder tumor, papillary urothelial carcinoma in the bladder was diagnosed. Normal concentrations of catecholamines were observed as shown in Table 1. His father had been dead for kidney cancer, while both his mother and his younger sister (31 years old) are healthy. Furthermore, his two sons (10 and 5 years old, respectively) are healthy in recent hospital examination (10 March 2021).

Through WES of the genomic DNA of proband 2, a recurrent *VHL* variant, c.558_560AGAdel, in exon 3 was found with the amino acid glutamic acid in codon 186 (GAA) deleted, which has not been recorded in the dbSNP database (Figure 2B). We had not found this variant in the database of gnomAD or CMDB, but it had been recorded in HGMD. The variant c.558_560AGAdel was verified by the Sanger sequencing (Figures 2C, D). The two sons and sister of proband 2 were sequenced for the c.558_560AGAdel variant by Sanger sequencing; only his older son carried this variant. In addition, this variant was predicted to be deleterious with a PROVRAN score of −8.98 and MutationTaster also demonstrated it to be disease causing. Alignment of VHL protein sequences with the Clustal Omega program demonstrated that the glutamic acid at codon 186 of the VHL protein was conserved (Figure 2E).

### Proband 3

**FIGURE 3:**
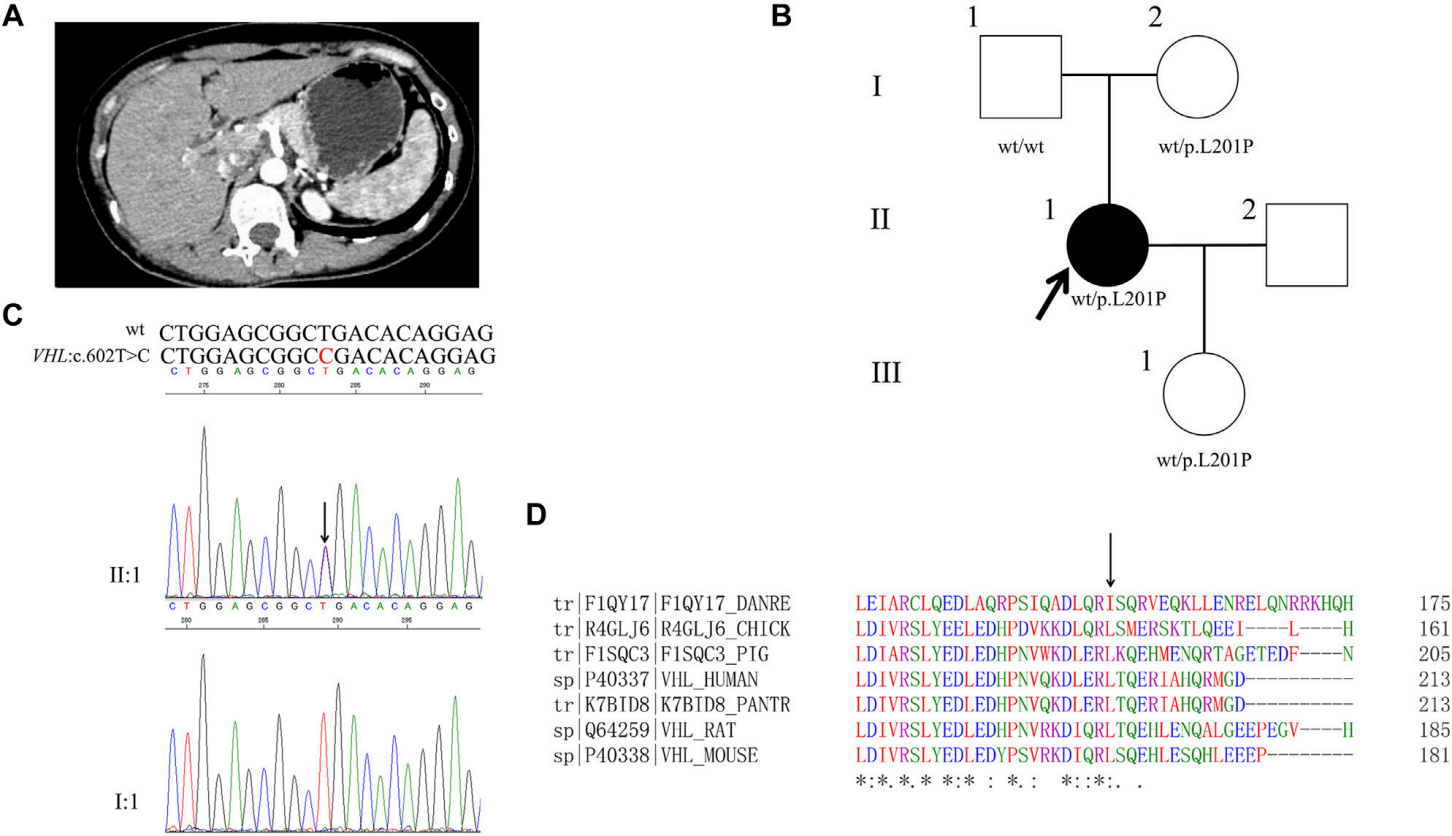
Clinical and genetic characteristics of Proband 3. **(A)** Postoperative contrast-enhanced CT scan of adrenal in a 36-year-old female patient with PPGL. **(B)** Pedigree of the Proband 3 family with PPGL. Squares and circles indicate males and females, respectively; Roman numerals indicate generations; and Arabic numerals indicate individual family members. Each member’s *VHL* genotype are shown below the squares and circles. Wt, wild type. L, lysine; P, proline. **(C)** DNA sequencing data of an unaffected male (I:1) and the Proband 3 (II:1) with the heterozygous variant of *VHL* c.602T>C. wt, wild type. **(D)** Multiple protein sequence alignment of seven species for VHL p. L201 (arrow). An asterisk (*) indicates positions that are 100% conserved in the alignment, and a colon (:) indicates conservation between groups of strongly similar properties, whilst a period (.) indicates conservation between groups of weakly similar properties. The residues in the alignment are coloured to indicate their physicochemical properties. Red indicates small and hydrophobic amino acids, blue indicates acidic residues, magenta indicates basic and green denotes hydroxyl/sulfhydryl/amine residues.

A 36-year-old female was hospitalized with complaints of hypertension. She had been performed for the right adrenalectomy when she was 8 years old and the left adrenalectomy when she was 31 years old (Figure 3A). Normal concentrations of catecholamines except a slightly higher concentration of plasma 5-hydroxytryptamine were observed as shown in Table 1.

Through WES of the genomic DNA of proband 3, a novel *VHL* variant, c.602T>C, in exon 3 was found with the amino acid leucine in codon 201 (CTG to CGG) changed to proline, which has not been recorded in the dbSNP database (Figure 3B). We had not found this variant in the database of gnomAD, CMDB or HGMD. By the Sanger sequencing, the variant c.602T>C was verified (Figure 3C). The parents and daughter of proband 3 were sequenced for the c.602T>C variant by Sanger sequencing; both her mother and daughter carried this variant. By PolyPhen-2, this variant was predicted to be probably damaging with a score of 1.00. In addition, this variant was predicted to be deleterious with a PROVRAN score of −3.95 and MutationTaster and SIFT also demonstrated it to be disease causing. Clustal Omega program demonstrated that the lysine at codon 201 of the VHL protein was conserved (Figure 3D).

### Proband 4

**FIGURE 4:**
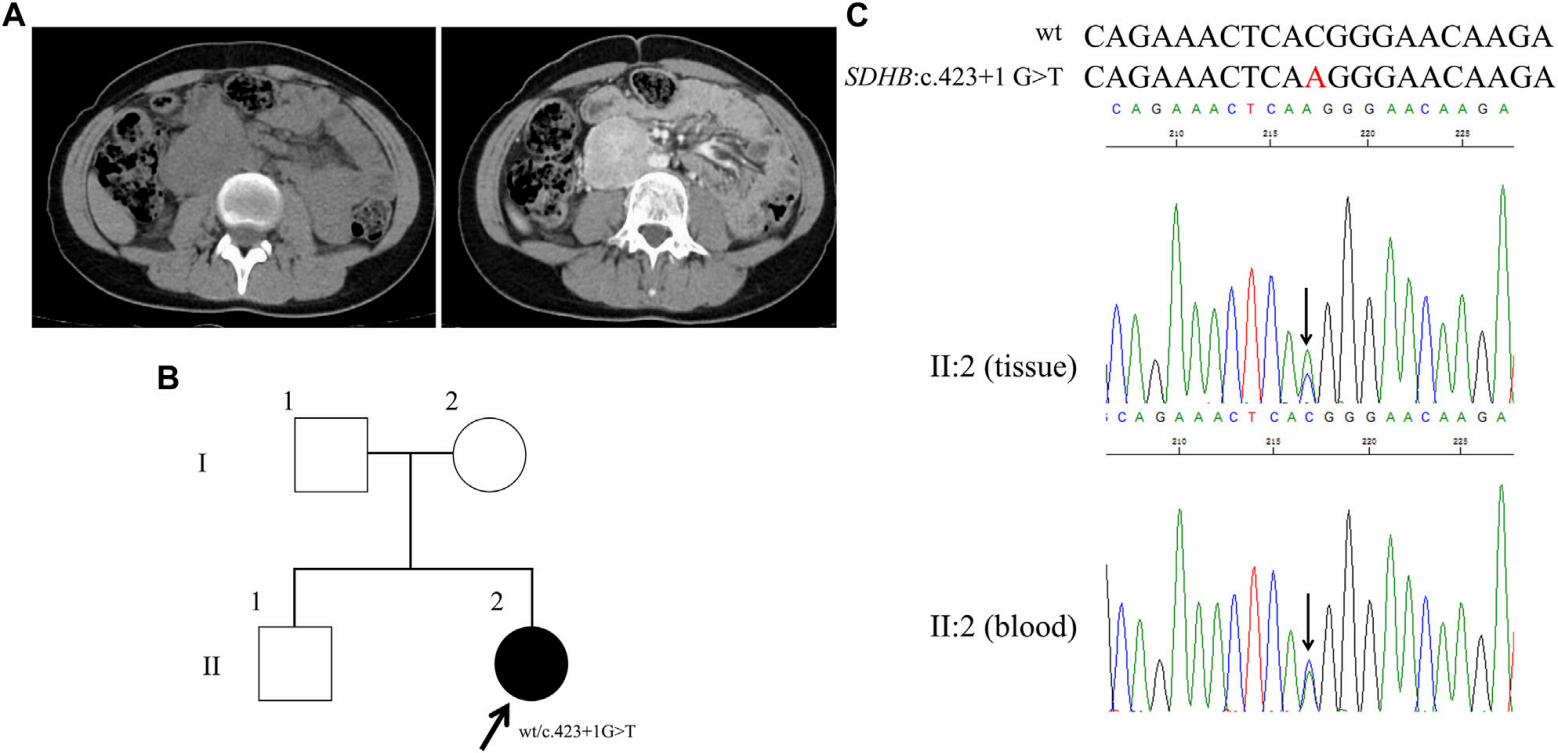
Clinical and genetic characteristics of Proband 4. **(A)** CT (left) and contrast-enhanced CT scan (right) imaging in a 17-year-old female patient with PPGL showing a right retroperitoneal occupancy. **(B)** Pedigree of the Proband 4 (adopted by the family) family with PPGL. Squares and circles indicate males and females, respectively; Roman numerals indicate generations; and Arabic numerals indicate individual family members. Each member’s *SDHB* genotype are shown below the squares and circles. Wt, wild type. **(C)** DNA sequencing data of the Proband 4 (II:2, tissue, and blood) with the heterozygous variant of *SDHB* c.423 + 1 G > T. Wt, wild type.

A 17-year-old girl was hospitalized with complaints of episode headache, profuse sweat and palpitation for 4 years. Higher concentrations of catecholamines were observed as shown in Table 1. Through abdominal CT, a right retroperitoneal occupancy was found but no occupancy was found in adrenal (Figure 4A). After an operation, right retroperitoneal paraganglioma was diagnosed by the immunohistochemistry.

By WES for the genomic DNA from the paraganglioma tissue of the proband 4, we identified a genetic variant in *SDHB* (succinate dehydrogenase complex iron sulfur subunit B), S*DHB*: NM_003000: exon4: c.423 + 1 G>T, which has not been recorded in the dbSNP database (Figure 4B). Sanger sequencing of the genomic DNA from both the paraganglioma tissue and blood of the proband verified this variant (Figure 4C). This variant was a splice site mutation in intron 4. By MutationTaster and CADD this variant was demonstrated to be disease causing. This variant has not been recorded in ClinVar or in CMDB. Totally four missense and one stop gained variants of *SDHB* gene were found in CMDB, which were all rare with a minor allele frequency less than 0.01 (Supplementary Table S2).

### Proband 5

**FIGURE 5:**
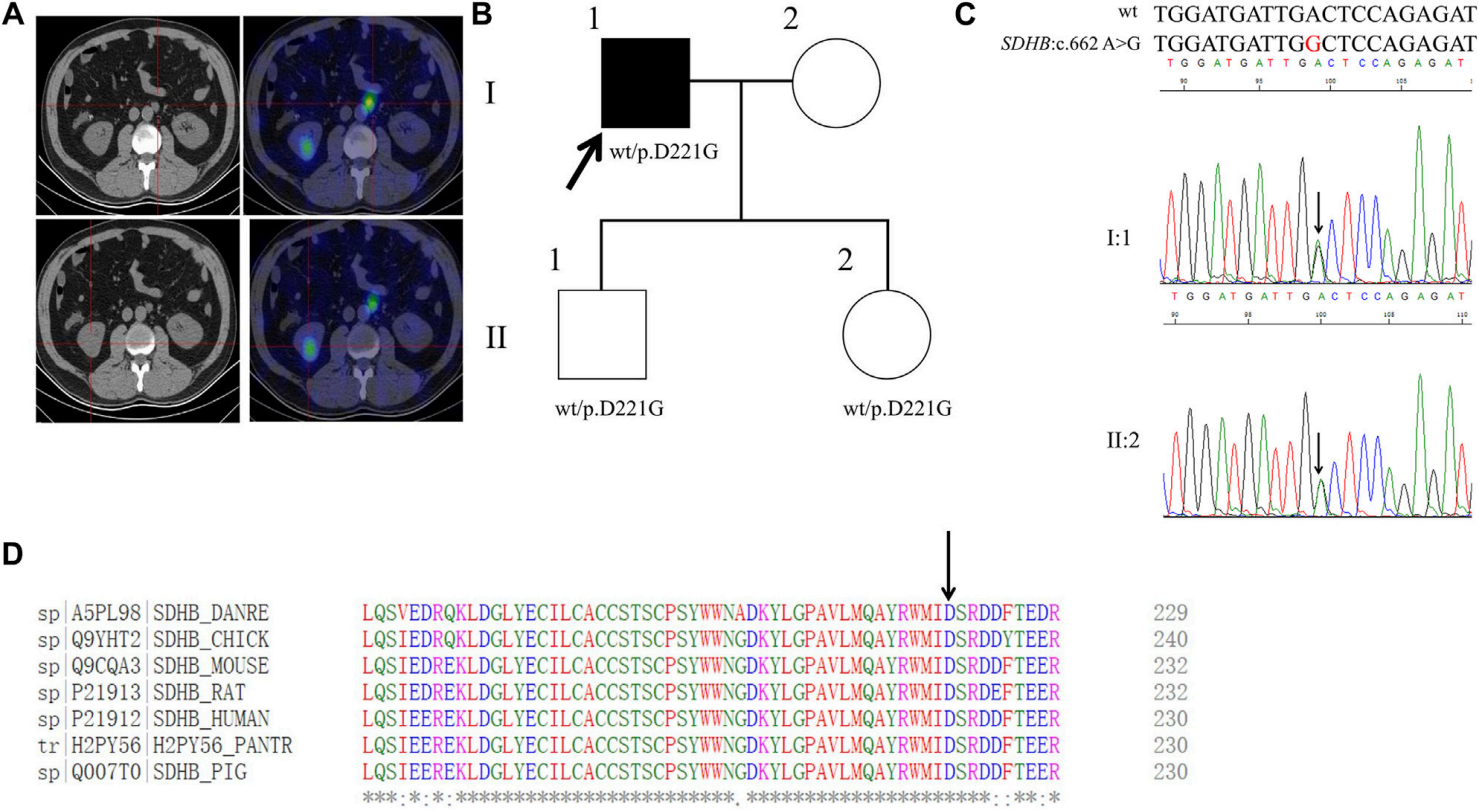
Clinical and genetic characteristics of Proband 5. **(A)** 131I-MIBG SPECT/CT imaging in a 53-year-old male patient with PPGL had shown a right kidney mass and a left retroperitoneal occupancy. CT (left) and SPECT/CT (right) transaxial sections are shown for the left retroperitoneal occupancy (upper) and the right kidney mass (bottom). **(B)** Pedigree of the Proband 5 family with PPGL. Squares and circles indicate males and females, respectively; Roman numerals indicate generations; and Arabic numerals indicate individual family members. Each member’s *SDHB* genotype are shown below the squares and circles. Wt, wild type. D, aspartic acid. G, glycine. **(C)** DNA sequencing data of the Proband 5 (I:1) and his daughter (II:2) with the heterozygous variant of *SDHB*:c.662 A>G. wt, wild type. **(D)** Multiple protein sequence alignment of seven species for SDHB p.D221 (arrow). An asterisk (*) indicates positions that are 100% conserved in the alignment, and a colon (:) indicates conservation between groups of strongly similar properties, whilst a period (.) indicates conservation between groups of weakly similar properties. The residues in the alignment are coloured to indicate their physicochemical properties. Red indicates small and hydrophobic amino acids, blue indicates acidic residues, magenta indicates basic and green denotes hydroxyl/sulfhydryl/amine residues.

A 53-year-old male had been diagnosed as hypertension for 12 years. Two years ago, abdominal CT showed a right adrenal occupancy and right pheochromocytoma was diagnosed. One year later after the operation, an occupancy in the inferior pole of the proband’s right kidney and a left retroperitoneal occupancy were found through abdominal CT (Figure 5A). Right papillary renal cell carcinoma and left retroperitoneal pheochromocytoma were diagnosed. Higher concentrations of catecholamines were detected as shown in Table 1.

By WES for the genomic DNA from the pheochromocytoma tissue of the proband 5, we identified a genetic variant in *SDHB*, SDHB: NM_003000:exon7:c.662A>G:p. D221G (the codon 221 of GAC to GGC), which has not been recorded in the dbSNP database (Figure 5B). Sanger sequencing of the genomic DNA from both the pheochromocytoma tissue and blood of the proband verified this variant (Figure 5C). By PolyPhen-2, this variant was predicted to be probably damaging with a score of 1.00. In addition, this variant was predicted to be deleterious with a PROVRAN score of −5.996 and MutationTaster and SIFT also demonstrated it to be disease causing. Clustal Omega program demonstrated that the aspartic acid at codon 221 of the SDHB protein is conserved among diverse species (Figure 5D). By Sanger sequencing, we identified this variant in the proband’s daughter and son. This variant has not been recorded in ClinVar or in CMDB.

### Proband 6

**FIGURE 6:**
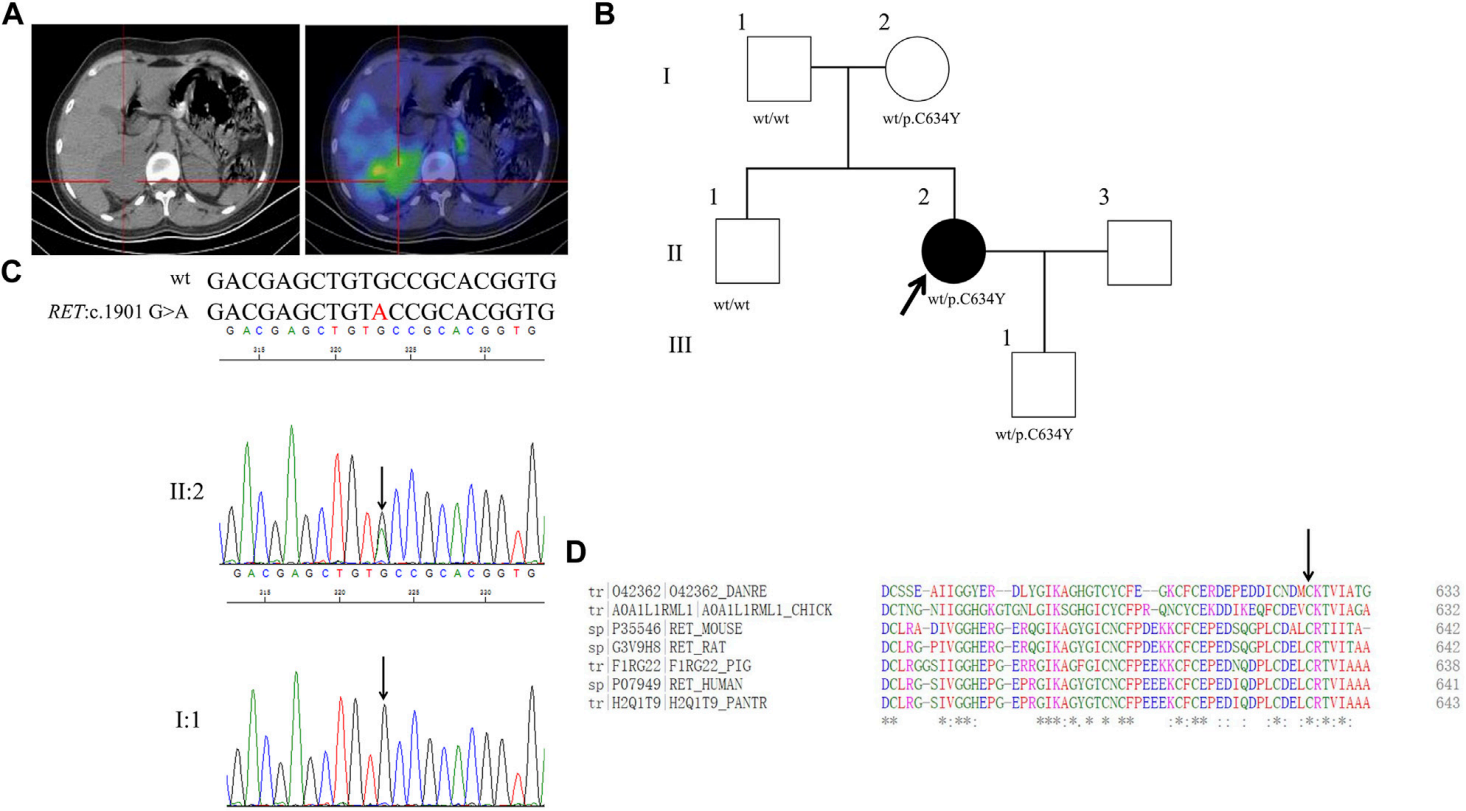
Clinical and genetic characteristics of Proband 6. **(A)** 131I-MIBG SPECT/CT imaging in a 27-year-old female patient with PPGL showing a right adrenal occupancy. CT (left) and SPECT/CT (right) transaxial sections are shown for the right adrenal occupancy. **(B)** Pedigree of the Proband 6 family with PPGL. Squares and circles indicate males and females, respectively; Roman numerals indicate generations; and Arabic numerals indicate individual family members. Each member’s *RET* genotype is shown below the squares and circles. Wt, wild type. C, cystein. Y, tyrosine. **(C)** DNA sequencing data of an unaffected male (I:1) and the Proband 6 (II:2) with the heterozygous variant of *RET*:c.1901 G>A. wt, wild type. **(D)** Multiple protein sequence alignment of seven species for RET p.C634 (arrow). An asterisk (*) indicates positions that are 100% conserved in the alignment, and a colon (:) indicates conservation between groups of strongly similar properties, whilst a period (.) indicates conservation between groups of weakly similar properties. The residues in the alignment are coloured to indicate their physicochemical properties. Red indicates small and hydrophobic amino acids, blue indicates acidic residues, magenta indicates basic and green denotes hydroxyl/sulfhydryl/amine residues.

A 27-year-old female has been diagnosed as hypertensive for 3 years. Abdominal enhanced CT found that she had an occupancy in the right adrenal and enlargement of left adrenal and ^131^I-MIBG was positive for the patient’s right adrenal (Figure 6A). Higher concentrations of catecholamines were observed as shown in Table 1. Pheochromocytoma was identified.

Through WES for the genomic DNA from the pheochromocytoma tissue of the proband 6, we identified a genetic variant in *RET* (ret proto-oncogene),RET:NM_020975:exon11:c.1901G>A:p.C634Y (the codon 634 of TGC to TAC), which has a dbSNP number of rs75996173 (Figure 6B). Sanger sequencing of the genomic DNA from both the pheochromocytoma tissue and blood of the proband verified this variant (Figure 6C). By PolyPhen-2, this variant was predicted to be probably damaging with a score of 1.00. In addition, this variant was predicted to be deleterious with a PROVRAN score of—6.953 and MutationTaster and SIFT also demonstrated it to be disease causing. Aligning the RET protein sequences with the Clustal Omega program demonstrated that the cysteine 634 in RET protein is conserved among diverse species (Figure 6D). By Sanger sequencing, we identified *RET* p.C634Y in the proband’s mother and son, but not in her father or brother. This variant had been recorded in ClinVar but not in CMDB. Totally twenty-four missense variants of *RET* were found in CMDB (Supplementary Table S2).

## Discussion

In this study, we explored the clinical details and genotypes of six Chinese patients with PPGL. We discovered one new *VHL* missense variant, and two new *SDHB* variants (one splice donor variation and one missense), and two recurrent variants in *VHL* and one in *RET*.

As the wide use of clinical imaging techniques including CT and MRI in patients and/or healthy individuals with medical examination, PPGL has shown a 4.8-fold increasing incidence from 1.4 per million people-years in 1977 to 6.6 in 2015 (Granberg et al., 2021). Genetic testing of susceptibility genes could confirm the diagnosis of PPGL and assist in further therapy, especially for VHL disease. In our proband 1 (VHL disease type 2A, PCC with low risk of renal cell carcinomas), proband 2 (VHL disease type 2B, PCC with high risk of renal cell carcinomas), and proband 3 (VHL disease type 2C, PCC without any other carcinomas), the amino acid residues 95, 186, and 201 of VHL protein with substitution or deletion were identified, respectively. The proline 95 and lysine 201 are located in the *ß* Domain of VHL, which binds HIF for its subsequent degradation by the ubiquitination-proteasome system (Shen and Kaelin, 2013). The variants p.P95R and p.L201P of VHL disrupt its binding with HIF and result in accumulation of HIF, which activates the transcription of target genes of HIF and the pathogenesis of tumors. The glutamic acid 186 locates in the *a* Domain of VHL, which binds Elongin C, Elongin B, Cullin 2 and Rbx1 to constitute the E3 ubiquitin protein ligase complex. The variant p.186Edel of VHL protein affects the generation of E3 ligase complex but not the binding with HIF.

About 40% of paragangliomas and 3% of pheochromocytomas are associated with deficiency of SDH, which plays a vital role linking the Krebs cycle and the electron transport chain as a respiratory enzyme (Gill et al., 2010). SDH includes four subunits, SDHA, SDHB, SDHC, and SDHD, named SDHx as a whole and forming the respiratory complex II. Two highly conserved L(I)YR motifs of SDHB form the Fe-S center of complex II, which is necessary for the electron transfer from dehydrogenase of succinate into fumatate (Jochmanova et al., 2017). PPGL patients with a *SDHB* variant are more prone to be malignant and metastatic. The proband 4 with a retroperitoneal paraganglioma has a splice donor variant of *SDHB*, c.423 + 1 G>T, and the proband 5 with a pheochromocytoma and renal cell carcinoma has a missense variant of *SDHB*, c.662A>G (p. D221G). These two single nucleotide variations have not been reported previously, to our knowledge. Both tissue and peripheral blood from proband 4 harbored the variant c.423 + 1 G>T of *SDHB*, which suggested its germline origination. However, the pedigree analysis for the proband 4 was not available because of her adoption by the family. The son and daughter of the proband 5 inherited the variant c.662A>G (p. D221G) of *SDHB* from him but were healthy when the study was performed.

The decreasing cost of next-generation sequencing technique made it affordable for most PPGL patients. More novel genetic variants of the diverse susceptibility genes were found in Korean, Japanese, Singaporean, Arab populations other than Europeans (Choi et al., 2020; Faiyaz-Ul-Haque et al., 2020; Ting et al., 2020; Yonamine et al., 2021). Different clinical manifestations and genetic spectrum of the PPGL susceptibility genes, especially *VHL*, *SDHB*, and *RET*, had been identified. The latest advances in the known genetics of neuroendocrine tumors such as *SUCLG2* (succinyl-CoA ligase *ß* Subunit G2, an oxygen-sensing domain in hypoxia inducible factor 2) and *IDH2* (isocitrate dehydrogenase 2) were recently reported (Lang et al., 2021; Hadrava Vanova et al., 2022). Because some probands showed no family history, which raised concerns that these mutations might be passenger mutations but not disease drivers. However, driver mutations are usually the same in different patients with the same cancer, but passengers are all different. WES analysis of our probands made the identified variants strong evidence for the causing of PPGL. Further functional tests would extending our understanding of the genetic variants found in the present study. Discrepancies among world populations confirmed the phenotype and genetic heterogeneity of PPGL, which should be considered in the diagnosis and therapeutics.

In summary, we explored the phenotype and genotype details in six Chinese PPGL patients and their relatives and identified three novel genetic variants in *VHL* (c.602T>C, p.L201P) and *SDHB* (c.423 + 1G>T and c.662A>G, p.D221G) genes with the potential to assist in diagnosis and treatment of PPGL in the future.

## Data Availability

The datasets presented in this study can be found in online repositories. The names of the repository/repositories and accession number(s) can be found in the article/supplementary material.
