# Supplementary material for: Novel and recurrent genetic variants of VHL, SDHB, and RET genes in Chinese pheochromocytoma and paraganglioma patients
Source: Front Genet. 2023 Mar 3;14:959989. doi: 10.3389/fgene.2023.959989 (PMC10020357; doi:10.3389/fgene.2023.959989)
Supplement: Supplementary file 2 [file Table1.DOC]

Supplementary Table S1

The symptoms and characterization differences between VHL and SDH complex

| **Proband** | **VHL** | **SDH complex** |
| --- | --- | --- |
| Mutant Gene | *VHL* | *SDHB*, *SDHC* and *SDHD* genes |
| Genotype | TSG | TSG |
| Hereditary Mode | AD | SDHB:AD  SDHC:AD  SDHD:AD; PT |
| Onset Age | 30 years (5-58) | 31.7 years (3-75) |
| Tumor Site | PCC (Bil PCC50%)＞＞＞TA, HN | SDHB:TA＞HN＞PCC  SDHC:HN(especially CBP)＞TA＞PCC  SDHD:HN＞TA＞PCC |
| Produced Hormones | metanephrines (MNs) | catecholamine |
| Symptoms | hypertension, tachycardia and palpitations | asymptomatic and inactive |
| Metastasis Risk | low(＜5%) | SDHB: high risk for metastasis and recurrence (30%-70%)  SDHC: low  SDHD: low but can extend into the skull |
| Compositions of Syndrome | CNS hemangioblastoma, renal or pancreatic cysts, renal carcinoma and exodermic cystadenoma. | renal cell carcinoma, gastrointestinal stromal tumor, pituitary adenoma |
| Penetrance | 50%-97% | SDHB: 8%-37%  SDHC: 8%  SDHD: 38%-64% |

TSG, tumor suppressor gene; AD, autosomal dominant inheritance; PT, autosomal dominant, paternal inheritance (maternal imprinting); PCC, pheochromocytoma; Bil, bilateral; TA, thoracoabdominal; CBP, carotid body paraganglioma; HN, head and neck paraganglioma;
